# A cultured strain of "Helicobacter heilmannii," a human gastric pathogen, identified as H. bizzozeronii: evidence for zoonotic potential of Helicobacter.

**DOI:** 10.3201/eid0706.010622

**Published:** 2001

**Authors:** K Jalava, S L On, C S Harrington, L P Andersen, M L Hänninen, P Vandamme

**Affiliations:** Department of Food and Environmental Hygiene, Faculty of Veterinary Medicine, University of Helsinki, Finland. katri.jalava@ktl.fi

## Abstract

We compared the characteristics of a cultured human "Helicobacter heilmannii" isolate with those of other helicobacters found in animals. Phenotypic, protein profile, 16S rDNA sequence, and DNA-DNA hybridization analyses identified the human strain as H. bizzozeronii, a species frequently found in dogs. Thus, H. bizzozeronii may have zoonotic potential.


### A Cultured Strain of "*Helicobacter heilmannii*," a Human Gastric Pathogen, Identified as *H. bizzozeronii*: Evidence for Zoonotic Potential of *Helicobacter*

**Katri Jalava,* Stephen L.W. On,† Clare S. Harrington,†**
**Leif P. Andersen,‡ Marja-Liisa Hänninen,* and Peter Vandamme§**
*Faculty of Veterinary Medicine, University of Helsinki, Finland; †Danish Veterinary Laboratory, Copenhagen, Denmark; ‡Rigshospitalet, Copenhagen, Denmark; and §University of Ghent, Ghent, Belgium

We compared the characteristics of a cultured human "*Helicobacter heilmannii*" isolate with those of other helicobacters found in animals. Phenotypic, protein profile, 16S rDNA sequence, and DNA-DNA hybridization analyses identified the human strain as *H. bizzozeronii*, a species frequently found in dogs. Thus, *H. bizzozeronii* may have zoonotic potential.

The importance of *Helicobacter pylori* in human gastric disease is well recognized (1). After its initial isolation, a distinctive, tightly coiled organism was also observed in some human gastritis cases (2). Although these organisms could not be isolated in vitro (3,4), they were designated as "*Gastrospirillum hominis*" on the basis of morphology and ecologic niche (4), and later as "*H. heilmannii*" on the basis of 16S rRNA gene sequence analysis. Differences in 16S rRNA gene sequences have allowed two types to be defined for "*H.heilmannii*," which accounts for 0.2% to 4% of human gastritis cases (5). Evidence suggests that infections with this organism may be more frequently associated with mucosa-associated lymphoid tissue lymphomas (6).

Animals, especially cats, dogs, and pigs, are frequently infected with large spiral organisms (often referred to collectively as gastrospirilla) that closely resemble "*H. heilmannii*" strains in humans (3,7-10). As a consequence, the possibility of zoonotic infection has been considered (3,10,11). Three distinct species resembling "*H. heilmannii*" have been isolated from cats or dogs, namely *H. felis, H. bizzozeronii,* and *H.salomonis* (10). The provisional name "*Candidatus* H. suis" has been proposed for the pig organism (8). However, critical comparison of strains from each of these sources has not been possible because few workers have obtained cultures of human strains. To our knowledge, only one "*H. heilmannii*" strain has been cultured from human tissue (6).

We present the results of a polyphasic identification analysis that confirms this human "*H. heilmannii*" isolate as *H. bizzozeronii*, and we discuss the public health implications of this finding.

### The Study

The human "*H. heilmannii*" isolate, Rigshospitalet 53 (R-53), and other *Helicobacter* strains for comparison were cultured by using conditions described earlier for phenotypic (12), whole-cell protein (13), 16S rRNA sequence (12), and DNA-DNA hybridization (10) analyses. A total of 66 phenotypic tests were determined for R-53, and results were compared both empirically and by numerical analysis with similar data for 116 other strains of *Helicobacter*, as described (12). Primers and methods used for polymerase chain reaction (PCR) amplification and direct sequencing of the full 16S rRNA gene were as described previously, as were the methods used for sequence alignment and phylogenetic analysis (14). The whole genomic DNA was isolated and dot-blot DNA-DNA hybridizations were performed (10), with DNA derived from R-53 used as a probe to characterize the type strains of *H. felis, H. bizzozeronii*, and *H. salomonis*. Whole-cell protein samples were prepared, separated by polyacrylamide gel electrophoresis (running length 9 cm), digitized, and subjected to comparative numerical analysis(13).

The phenotypic characteristics of R-53 were consistent with those documented for *H. felis, H. bizzozeronii*, and *H.salomonis* (10), with two principal differences: the cultures formed distinct colonies, and the growth exhibited was profuse with a greasy surface film. This morphology is typical of *H. pylori* (1) but differs from the sparse growth lacking defined colony formation observed in cultures of the other *Helicobacter* species mentioned (10). Furthermore, R-53 grew weakly on 1.0% and 1.5% ox-bile media. This trait is rare among gastric helicobacters but more common in species colonizing the lower intestine (15). Nonetheless, numerical comparison of phenotypic characters demonstrated that R-53 was most similar (90.9%) to a reference strain (Wiberg) of *H. bizzozeronii*. These two strains formed part of a larger cluster delineated at the 87% similarity level containing all 12 *H. bizzozeronii* and six *H. salomonis* isolates studied, as well as 12 of 15 *H. felis* and an *H. muridarum* strain (a mouse isolate). This cluster was clearly differentiated from all other *Helicobacter* species, including 14 type and reference strains of *H. pylori* obtained from humans, monkeys, and a pig (data not shown).

Address for correspondence: Katri Jalava, Department of Infectious Diseases Epidemiology, National Public Health Institute, Mannerheimintie 166, FIN-00300 Helsinki, Finland; fax: 358-9-4744 8468; e-mail: katri.jalava@ktl.fi

A phylogenetic tree based on comparisons of 16S rRNA gene sequences confirmed previous observations (6) that R-53, *H. felis, H. bizzozeronii, H. salomonis*, and "*H. heilmannii*" type 2 were highly related and formed a distinct cluster within the rRNA homology group III (i.e., the *Helicobacte*r phylogenetic branch) of rRNA superfamily VI (data not shown). Although the infra- and intraspecific sequence variation observed among these species makes unequivocal differentiation between them impossible, these species are readily distinguished from other helicobacters, including the "*H.heilmannii*" type 1 taxon. The closest phylogenetic neighbor of the latter is "*Candidatus* H. suis" (8), which may be found in up to 87% of pigs (7). The complex relationships observed within the "*H. heilmannii*" type 2-containing clade exemplify cases in which 16S rDNA sequence comparisons cannot be used as an accurate species identification method, as also seen with the related taxa *H. cinaedi* (16) and *C. hyointestinalis* (14).

Numerical comparison of protein profiles of R-53 with those of all extant *Helicobacter* species placed this strain in a cluster with type and reference strains of *H. bizzozeronii* (Figure 1). This clustering confirms the earlier finding that species identification of cultured *Helicobacter* strains is efficiently performed with highly standardized whole-cell protein analysis, and the results are in excellent agreement with those of DNA-DNA hybridization (10,13,16). Dot-blot DNA-DNA hybridization demonstrated that the R-53 probe did not hybridize intensively with genomic DNA of either *H. felis* or *H. salomonis*, but hybridized strongly with DNA from all isolates of *H. bizzozeronii*, including homologous assays (Figure 2). Therefore, compilation of the phenotypic and genotypic data determined for R-53 clearly identify it as a strain of *H. bizzozeronii*, the most common *Helicobacter* species (approximately 44% of isolates) found in healthy and diseased pet dogs (10).

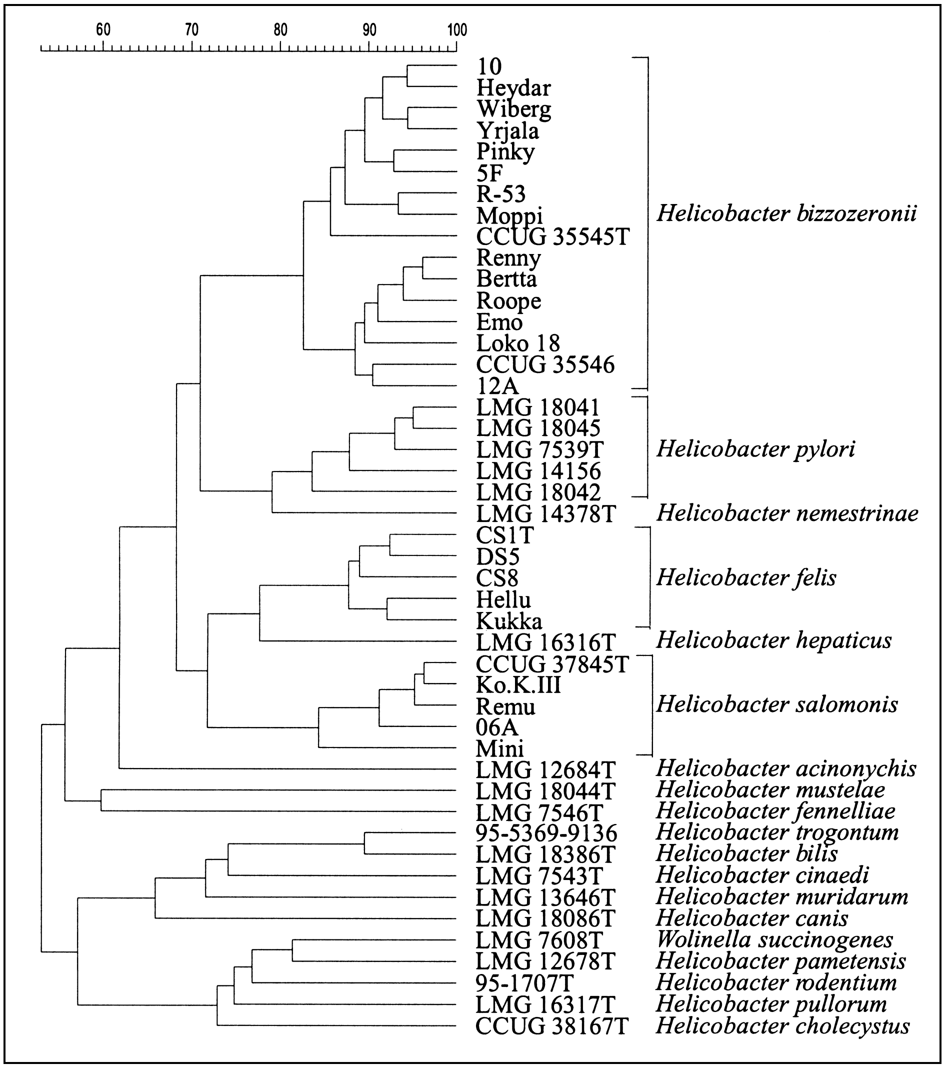

Figure 1. Dendrogram expressing similarity in whole-cell protein patterns of strain R-53, and type and reference strains of all *Helicobacter* and *Wolinella* species.

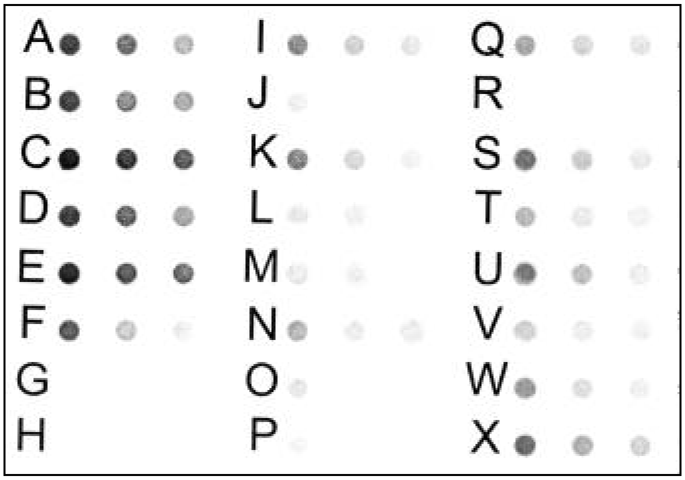

Figure 2. Dot-blot DNA-DNA hybridization assay with R-53 as a probe. A-F *Helicobacter bizzozeronii* strains: A. R-53, B. CCUG 35545 $^{T}$, C. CCUG 35546, D. 12A, E. 10, F. Poju; G-M *H. felis* strains: G. CS1$^{T}$, H. DS3, I. CCUG 37471, J. 15/390, K. Dog 1, L. Dog 3, M. CS2; N-P *H. pylori* strains: N. Tx30, O. 26695, P. CCUG 17874$^{T}$; Q. *H. mustelae* CCUG 25715$^{T}$; R. *H. acinonychis* CCUG 29263$^{T}$; S-X *H. salomonis* strains: S. 06A, T. CCUG 37845$^{T}$, U. Vilho, V. Mini, W. Remu, X. Ko.K. III.

### Conclusions

Although further studies are needed to clarify the taxonomic relationships between "*H. heilmannii*" type 1, "*Candidatus* H. suis," and members of the *H. felis-bizzozeronii-salomonis* species complex, available data strongly indicate that human infections with gastrospirilla may be acquired from more than one source, including pigs, dogs, and cats. Several epidemiologic studies have shown an increased incidence of "*H. heilmannii*" in humans associated with animal contacts and these bacteria (11,17). PCR-restriction fragment-length polymorphism (RFLP) analysis of the *Helicobacter ureB* gene performed directly on stomach biopsy tissue showed the presence of multiple types of gastrospirilla in a single patient; the same PCR-RFLP type was also identified in the patient's cat (3). In conclusion, this study clearly demonstrates the zoonotic potential of large, spiral gastric *Helicobacter* spp. The only human "*H. heilmannii*" isolate to date was identified in a polyphasic taxonomic analysis as *H.bizzozeronii*. More studies with a number of isolates are needed to evaluate the full zoonotic potential of animal *Helicobacter* spp. Given the difficulties in culturing these organisms in vitro, available data strongly indicate the need for species-specific PCR assays for detecting the various *Helicobacter* spp. that inhabit the gastric mucosa of humans and animals.

### Acknowledgments

We thank Urszula Hirvi and Dirk Dewettinck for excellent technical assistance. We are grateful to all the depositors of the strains.

Katri Jalava has been supported by the Young Scientists Award of Emil Aaltosen Säätiö.

Dr. Jalava, whose research interests focus on the taxonomy of animal helicobacters, works in the National Public Health Institute of Finland. This study was carried out in the Department of Food and Environmental Hygiene of the University of Helsinki.

### References

1. Dunn BE, Cohen H, Blaser MJ. *Helicobacter pylori*. Clin Microbiol Rev 1997;10:720-41.
2. Dent JC, McNulty CA, Uff JC, Wilkinson SP, Gear MW. Spiral organisms in the gastric antrum [letter]. Lancet 1987;ii:96.
3. Dieterich C, Wiesel P, Neiger R, Blum A, Corthes y Theulaz I. Presence of multiple "*Helicobacter heilmannii*" strains in an individual suffering from ulcers and in his two cats. J Clin Microbiol 1998;36:1366-70.
4. McNulty CA, Dent JC, Curry A, Uff JS, Ford GA, Gear MW, et al. New spiral bacterium in gastric mucosa. J Clin Pathol 1989;42:585-91.
5. Solnick JV, O'Rourke J, Lee A, Paster BJ, Dewhirst FE, Tompkins LS. An uncultured gastric spiral organism is a newly identified Helicobacter in humans. J Infect Dis 1993;168:379-85.
6. Andersen LP, Boye K, Blom J, Holck S, Norgaard A, Elsborg L. Characterization of a culturable "*Gastrospirillum hominis*" (*Helicobacter heilmannii*) strain isolated from human gastric mucosa. J Clin Microbiol 1999;37:1069-76.
7. Cantet F, Magras C, Marais A, Federighi M, Mégraud F. *Helicobacter* species colonizing pig stomach: molecular characterization and determination of prevalence. Appl Environ Microbiol 1999;65:4672-6.
8. De Groote D, van Doorn LJ, Ducatelle R, Verschuuren A, Haesebrouck A, Quint WGV, et al. '*Candidatus* Helicobacter suis,' a gastric helicobacter from pigs, and its phylogenetic relatedness to other gastrospirilla. Int J Syst Bacteriol 1999;49:1769-77.
9. Jalava K, Kaartinen M, Utriainen M, Happonen I, Hanninen M-L. *Helicobacter salomonis* sp. nov., a canine gastric *Helicobacter* sp. related to *Helicobacter felis* and *Helicobacter bizzozeronii*. Int J Syst Bacteriol 1997;47:975-82.
10. Jalava K, On SLW, Vandamme PAR, Happonen I, Sukura A, Hanninen M-L. Isolation and identification of *Helicobacter* spp. from canine and feline gastric mucosa. Appl Environ Microbiol 1998;64:3998-4006.
11. Stolte M, Wellens E, Bethke B, Ritter M, Eidt H. *Helicobacter heilmannii* (formerly *Gastrospirillum hominis*) gastritis: an infection transmitted by animals? Scand J Gastroenterol 1994;29:106-14.
12. On SLW, Holmes B. Classification and identification of campylobacters, helicobacters and allied taxa by numerical analysis of phenotypic characters. System Appl Microbiol 1995;18:374-90.
13. Pot B, Vandamme P, Kersters K. Analysis of electrophoretic whole-organism protein fingerprints. In: Goodfellow M, O'Donnell AG, editors. Modern microbial methods. Chemical methods in prokaryotic systematics. Chichester (UK): John Wiley and Sons Ltd.; 1994. p. 493-521.
14. Harrington CS, On SLW. Extensive 16S rRNA gene sequence diversity in *Campylobacter hyointestinalis* strains: taxonomic and applied implications. Int J Syst Bacteriol 1999;49:1171-5.
15. On SLW, Holmes B, Sackin MJ. A probability matrix for the identification of campylobacters, helicobacters, and allied taxa. J Appl Bacteriol 1996;81:425-32.
16. Vandamme P, Harrington CS, Jalava K, On SLW. Misidentifying helicobacters: the *Helicobacter cinaedi* example. J Clin Microbiol 2000;38:2261-6
17. Meining A, Kroher G, Stolte M. Animal reservoirs in the transmission of *Helicobacter heilmannii*. Results of a questionnaire-based study. Scand J Gastroenterol 1998;33:795-8.
